# The influence of the specific growth rate on the lipid composition of *Sulfolobus acidocaldarius*

**DOI:** 10.1007/s00792-020-01165-1

**Published:** 2020-03-21

**Authors:** Julian Quehenberger, Ernst Pittenauer, Günter Allmaier, Oliver Spadiut

**Affiliations:** 1 Research Division Biochemical Engineering, Faculty of Technical Chemistry, Institute of Chemical, Environmental and Bioscience Engineering, TU Wien, Vienna, Austria; 2 Research Group for Mass Spectrometric Bio and Polymer Analytics, Faculty of Technical Chemistry, Institute of Chemical Technologies and Analytics, TU Wien, Vienna, Austria

**Keywords:** *Sulfolobus acidocaldarius*, Diether lipids, Tetraether lipids, Specific growth rate, Cyclopentane rings

## Abstract

Archaeal lipids are constituted of two isoprenoid chains connected via ether bonds to glycerol in the *sn*-2, 3 position. Due to these unique properties archaeal lipids are significantly more stable against high temperature, low pH, oxidation and enzymatic degradation than conventional lipids. Additionally, in members of the phylum Crenarchaeota condensation of two (monopolar) archaeal diether lipids to a single (bipolar) tetraether lipid as well as formation of cyclopentane rings in the isoprenoid core strongly reduce permeability of the crenarchaeal membranes. In this work we show that the Crenarchaeum *Sulfolobus acidocaldarius* changes its lipid composition as reaction to a shift in growth rate caused by nutrient limitation. We thereby identified a novel influencing factor for the lipid composition of *S. acidocaldarius* and were able to determine the effect of this factor on the lipid composition by using MALDI-MS for the semi-quantification of an archaeal lipidome: a shift in the specific growth rate during a controlled continuous cultivation of *S. acidocaldarius* from 0.011 to 0.035 h^−1^ led to a change in the ratio of diether to tetraether lipids from 1:3 to 1:5 and a decrease of the average number of cyclopentane rings from 5.1 to 4.6.

**Electronic supplementary material:**

The online version of this article (10.1007/s00792-020-01165-1) contains supplementary material, which is available to authorized users.

## Introduction

Although today Archaea are known to ubiquitously thrive in habitats all over the world, they have long been considered classical model organisms for “extreme” environments. This historic misconception might be a reason for the extensive interest in the archaeal membrane composition from very early on in archaeal research, as the membrane is the main protective barrier of a cell against its surroundings (van de Vossenberg et al. 1998). Indeed, the fundamental difference in membrane architecture and chemistry of membrane lipids between Archaea on the one hand and Bacteria and Eukarya on the other hand is a characteristic feature of distinction and probably the most obvious biochemical trait unique to the archaeal Domain. Archaeal membranes are composed of ether lipids: glycerol-1-phosphate backbones are linked via ether bonds to two isoprenoid chains (Koga and Morii 2007; Jain et al. 2014). Two main classes of these ether lipids dominate the archaeal lipid spectrum: polar diether lipids (DELs) commonly containing 20 or 25 carbon atoms per isoprenoid chain and monolayer forming bipolar tetraether lipids (TELs) commonly containing 40 carbon atoms per isoprenoid chain. The biosynthesis of DELs via the mevalonate pathway is well understood (Jain et al. 2014), while the mechanism of TEL synthesis is not resolved yet. Head-to-head condensation of two DELs and consecutive introduction of cyclic ring structures is the most popular hypothesis (Jain et al. 2014), while also a more complex, stepwise pathway has been proposed (Villanueva et al. 2014). Independent of the actual mechanism, two proteins, Saci_0421 and Saci_1201, responsible for the formation of cyclopentane in *Sulfolobusacidocaldarius* have been identified by Guan et al. (2017).

Described variations of the two main lipid structures include the following: (1) differences in head groups—a range of polar compounds and sugar residues were observed as head groups [e.g., phospholipids containing glycerol, serine, ethanolamine, myo-inositol or in case of glycolipids: glucose, galactose, mannose, gulose, *N*-acetylglucosamine or combinations thereof (Shimada et al. 2008; Oger and Cario 2013)]; (2) chain length of the isoprenoid core (e.g., Matsuno et al. 2009); (3) unsaturation (Dawson et al. 2012); (4) methylation of the isoprenoid chain (Knappy et al. 2009; Yoshinaga et al. 2015); (5) linkage between the isoprenoid chains (Morii et al. 1998); and (6) introduction of ring systems—generally ranging 1–8 cyclopentane rings (De Rosa and Gambacorta 1988; Lai et al. 2008; Jensen et al. 2015a), but also the incorporation of a hexane ring has been reported in multiple crenarchaeal species (Damsté et al. 2002, 2018).

As of now, most studies investigated the effect of growth temperature on the lipid composition. In general, Archaea react to increased temperatures with condensation of two monopolar to one single membrane-spanning bipolar lipid (e.g., Lai et al. 2008; Matsuno et al. 2009). As a further adaption strategy Crenarchaeota incorporate higher numbers of cyclopentane rings in their monopolar lipids (Chong 2010; Jensen et al. 2015b). Although no general correlation between the temperature optimum of a Crenarchaeum and the number of cyclopentane rings can be made, it is evident that a rise of the growth temperature stimulates the organisms to increase the number of rings in their lipids. In other archaeal phyla, in addition to the temperature, the influence of hydrostatic pressure, pH and salinity were investigated (e.g., Kaneshiro and Clark 1995; Shimada et al. 2008; Dawson et al. 2012). Clearly, when investigating the adaption of the lipid composition the focus in the scientific literature lies (1) on variations of these external, environmental factors (temperature, hydrostatic pressure, pH and salinity) and (2) on the comparison of exponential growth (log phase) vs. stationary phase which was done by many different groups with different organisms (e.g., Elling et al. 2014; Jensen et al. 2015b; Kramer and Sauer 1991; Matsuno et al. 2009; Meador et al. 2014; Morii and Koga 1993). A third factor with supposedly high potential to impact the lipid composition is (3) the level of energy and nutrient supply of the culture. A study performed by Bischof et al. (2019) showed that complete starvation of the uracil auxotrophic strain *S. acidocaldarius* MW001 resulted in a higher number of cyclopentane rings compared to nutrient rich conditions.

A factor that has never been tested for its influence on the lipid composition of Archaea is the specific growth rate (*µ*). In this study, we cultivated *S. acidocaldarius* DSM639, a member of the phylum Crenarchaeota and widely popular archaeal model organism, under nutrient limited conditions. We maintained two different substrate uptake rates (*q*_s_), and consequently two distinct µs, by employing different feeding rates during a continuous cultivation. Our goal was to determine the ratio of mono- to bipolar lipids (DEL:TEL ratio) and the number of cyclopentane rings in the isoprenoid lipid core in dependence of *µ* under controlled nutrient limiting conditions. Thereby, we aim to test the hypothesis that rather than a different growth phase, like already observed in previous studies, a change in the specific growth rate is the direct cause for changes in the lipid pattern of *S. acidocaldarius*.

## Materials and methods

### Cultivation and bioreactor setup

*S. acidocaldarius* DSM 639 was cultivated in a 3.6-L glass bioreactor (Labfors 3, Infors, Germany) implemented as continuous stirred-tank reactor (CSTR) with 2 L working volume. The preculture was grown in a 100-mL shake flask at 75 °C and 100 rpm in 50 mL VD-Medium (Quehenberger et al. 2019) containing 1.75 g/L Na-glutamate (MSG), 3 g/L d-glucose and 0.5 g/L citric acid, adjusted to pH 3.0 with sulfuric acid. At an OD_600_ of 0.75 the preculture was transferred aseptically to the culture vessel yielding an initial OD_600_ of 0.023 and a total starting volume for the initial batch phase of 1.5 L, containing 2 g/L MSG and 1 g/L d-glucose. Batch phase was performed at 75 °C, pH 3.1, followed by a short fed-batch phase to increase the working volume to 2.0 L. After reaching 2 L culture volume the vessel was operated in continuous mode by harvesting at the same rate as medium was being supplied via the substrate feed. To ensure the absence of time effects and to ensure reproducibility of the results, after performing a shift from low to high growth rate (*µ* = 0.011–0.035 h^−1^) we employed another phase of high dilution rate replicating the conditions of the initial phase (*µ* = 0.035 h^−1^). To ensure enough time for the organism to adapt to the different growth rates, sampling was performed at least 2 dwell times (~ 2.5 days and ~ 8 days, respectively) after changing the dilution rate.

Per sampling point 45 mL aliquots were withdrawn and cells were harvested via centrifugation (14,000*g*, 4 °C, 10 min). Sampling points and respective dry cell weights are shown in Table 1. The cell pellet was then stored at −20 °C for later use.

**Table 1:** End-conditions of the distinct phases of constant dilution rates

	Time at sampling (h)	Phase duration (h)	Dilution rate (*D*) and specific growth rate (*µ*) (h^−1^)	Dwell time (*τ*) (h)	Dry cell weight (DCW) (g/L)
Constant Phase 1	334.4	70.0	0.035	28.6	1.95 ± 0.05
Constant Phase 2	527.3	192.9	0.011	90.9	2.11 ± 0.12
Constant Phase 3	599.5	72.2	0.035	28.6	1.85 ± 0.13

Dissolved oxygen (dO_2_) was measured with a dissolved oxygen electrode Visiferm DO425 (Hamilton, USA) and dO_2_ levels were maintained above 30% by aerating with up to 0.6 L/min pressurized air. pH was monitored with an Easyferm electrode (Hamilton, USA) and kept at 3.1 by addition of H_2_SO_4_ (9.6% v/v) via the pump module of the bioreactor. Feed was supplied via a Preciflow peristaltic pump (Lambda, Switzerland) following a feed-forward controlled exponential feeding strategy, while broth was continuously withdrawn via a dip pipe and a Preciflow peristaltic pump (Lambda, Switzerland). Mixing was performed at 350 rpm. CO_2_ and O_2_ contents in the offgas were analyzed with BCP-O_2_ and BCP-CO_2_ gas analyzers (Bluesens, Germany). Process parameters were adjusted and recorded via the process information management system Lucullus (Securecell, Switzerland).

### Biomass determination

*Drycellweight*, *DCW *[g/L], was determined gravimetrically in triplicates. 5 mL fermentation broth were centrifuged (4000*g*, 4 °C, 15 min) and subsequently dried at 95 °C for at least 72 h.

*Opticaldensity*, *OD*_*600*_ [ ], was determined photometrically at 600 nm. Samples were diluted with deionized water to stay in the linear range of the photometer (Genesys 20, Thermo Fisher Scientific, USA).

### Calculation of dilution rate, specific growth rate and dwell time

*Dilutionrate*,* D* [1/h], in a CSTR was calculated as the quotient of volume flow [L/h] by the reactor volume [L].

*Specificgrowthrate*,* µ* [h^−1^], in an equilibrated CSTR can be assumed equal to *D*. Therefore, during the experimental phases *µ* was controlled and set to 0.11 and 0.35 h^−1^, respectively, via the volume flow, which is proportional to the feed rate [g/h], at a constant reactor volume.

*Dwelltime*,* τ* [h], was calculated as the reciprocal value of the dilution rate *D*.

### Lipid extraction

Frozen cell pellets of 45 mL culture broth per sample (dry cell weight at the time of harvest is shown in Table 1) were thawed and resuspended in 5 mL ammonium acetate (155 mM). After sonication on ice (6 × 1 min with 30 s cooling periods between pulses, duty cycle 50%, output control 7; Branson Sonifier 250, Thermo Fisher Scientific, USA) 20 mL of a chloroform/methanol solution (2:1 vol/vol) was added. Lipids were extracted via vigorous shaking on a vibrating panel at 2200 rpm (ika-vibrax-vxr Typ VX7, Janke & Kunkel, Germany) for 30 min at 4 °C. For phase separation the mixture was centrifuged (1000*g*, 4 °C, 2 min). Subsequently the lower organic phase was removed carefully and the solvent was evaporated to dryness under a nitrogen stream (0.2 L/min) at room temperature. The remaining solid lipid extract was stored at −20 °C for further analysis. All steps following the addition of chloroform/methanol were performed in glassware.

### Structural analysis of lipids

All measurements were performed using a Shimadzu MALDI 7090 tandem time-of-flight (TOF) mass spectrometer (Shimadzu-Kratos Analytical, Manchester, UK) described in detail elsewhere (Belgacem et al. 2016). Briefly, the instrument is fitted with a frequency-tripled 2 kHz Nd-YAG laser (*λ* = 355 nm) operated for sensitivity reasons at 200 Hz, a linear TOF analyzer as MS1 and a wide energy-acceptance curved field RTOF as MS2 for product ion analysis (close to 95% of the selected *m/z* range), a grounded gas collision cell with differential pumping of the collision region, a double Bradbury–Nielsen wire ion gate for precursor ion selection, an axial spatial distribution focusing (ASDF) lens for obtaining product ion resolutions up to 10,000 (FWHH) and two secondary electron multipliers for linear and reflectron TOF–MS, respectively, for ion detection.

All archaeal lipid samples were dissolved at roughly 1 mg per 1000 µL solvent (methanol:chloroform = 7:3 vol/vol). 2, 4, 6-Trihydroxyacetophenone was selected as MALDI-matrix for all experiments (positive- and negative-ion detection) by dissolving 15 mg matrix in 1000 µL methanol. Alternatively, the matrix solution was saturated with sodium chloride to improve mono- and disodiated molecules for positive-ion detection. For final sample preparation, analyte solution and matrix solution were mixed 1:1 (vol/vol) immediately before applying 1 µL of the resulting mixture onto the standard stainless steel target surface (dried droplet preparation).

All displayed positive- as well as negative-ion mass spectra represent an average of 10,000 unselected mass spectra (1 mass spectrum represents the average of 5 individual laser pulses). This number of shots was acquired in order to obtain satisfactory ion statistics across the mass range selected.

## Results and discussion

### Bioreactor cultivation

**Fig. 1:**
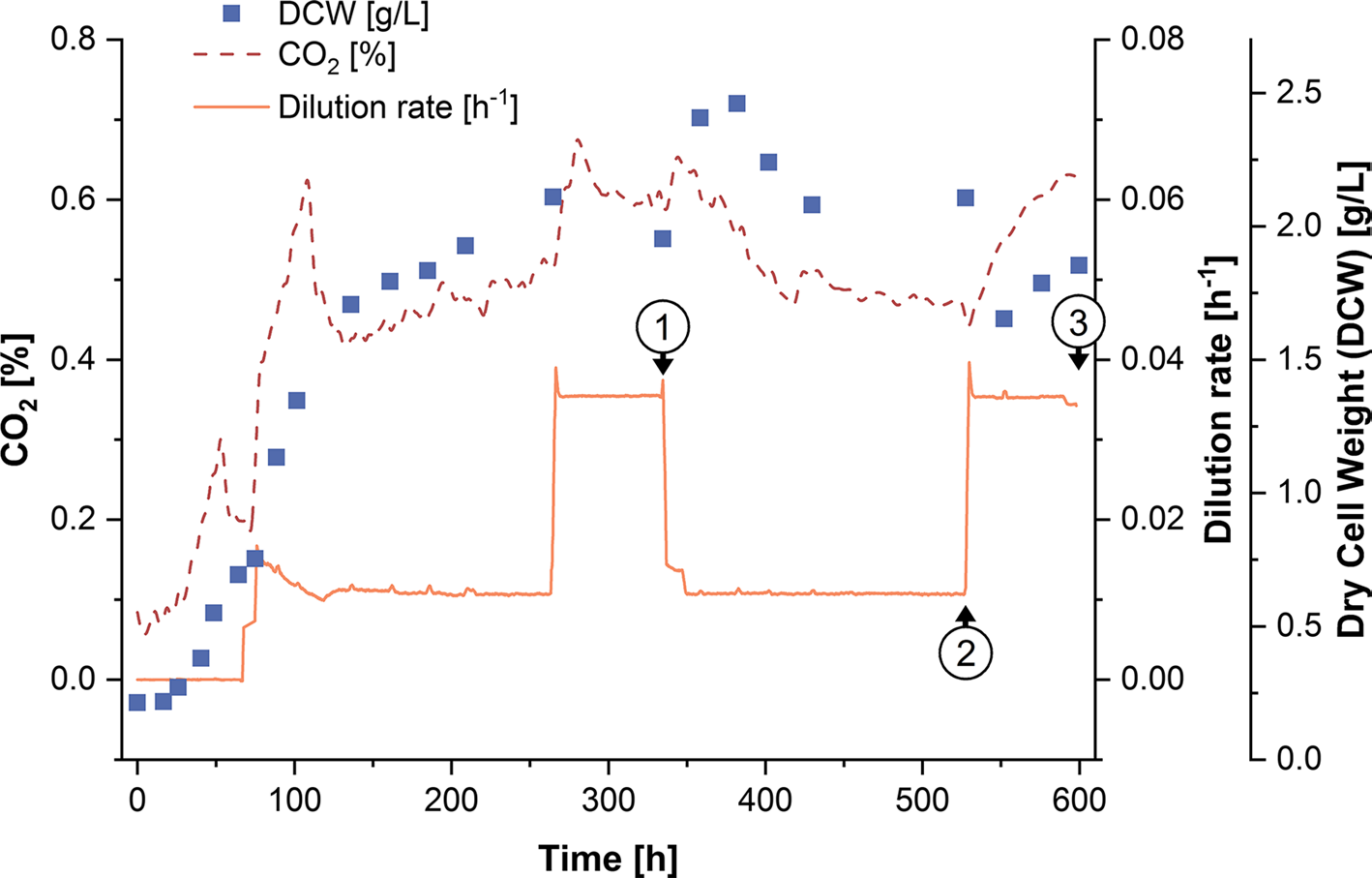
Continuous cultivation of *S. acidocaldarius* in a 3.6-L bioreactor (2.0 L working volume). Samples for determination of the lipid composition were drawn at the end of phases of constant dilution rate at the indicated time points 1, 2 and 3 (where 1 and 3 are replicates of the same biological state). Dry cell weight (DCW) [filled square], CO_2_ concentration [dashed line] and dilution rate (*D*) [horizontal line] are shown

Figure 1 shows the time course of the CO_2_ signal in the offgas which corresponds to the metabolic activity of the culture and the course of DCW which converges towards its equilibrium state. Dilution rate, calculated as the quotient of the feedrate by the reactor volume, is also shown in Fig. 1. DCW, *µ*, and *τ* at the end of each phase of constant *D*, as well as phase duration, are shown in Table 1. Owing to the increased dilution, the DCW is lower in the phases of higher *µ* (1.9 vs 2.1 g/L).

### Lipid composition and structure

In brief, like characteristic for members of the phylum Crenarchaeota, the lipids of *Sulfolobus* species consist of an isoprenoid core structure either in form of two diterpenoides or two tetraterpenoides connected with one or two glycerols, respectively, via ether bonds in the *sn*-2,3 positions. Polar head groups are connected in the *sn*-1 position and commonly consist of (poly-) hexoses (Hex), inositolphosphate (IP) or sulfonated trihexose (sulfono-Hex3). In the literature “archaeol”, “diether lipid” (DEL) or more specifically “dialkyl glycerol diether” (DGD) is common term for the diterpenoide containing C_40_ core structures, while the tetraterpenoide containing C_80_ core structures are known as “caldarchaeol”, “tetraether lipid” (TEL) or, more specifically, “glycerol dialkyl glycerol tetraether” (GDGT). These lipid classes which can be distinguished based on their lipid core and head groups can be further divided into lipid species. The latter describe lipids with the same head groups and number of carbon atoms, but with different number of cylopentane moieties. In this study for the notation of the lipid classes we follow the nomenclature described by Jensen et al. (2015a), using the terms DGD and GDGT for the description of the core structures preceded or followed with the above described abbreviations for the polar head groups. The chemical structures of the most abundant lipid classes found in this study are shown in the supplementary material.

**Fig. 2:**
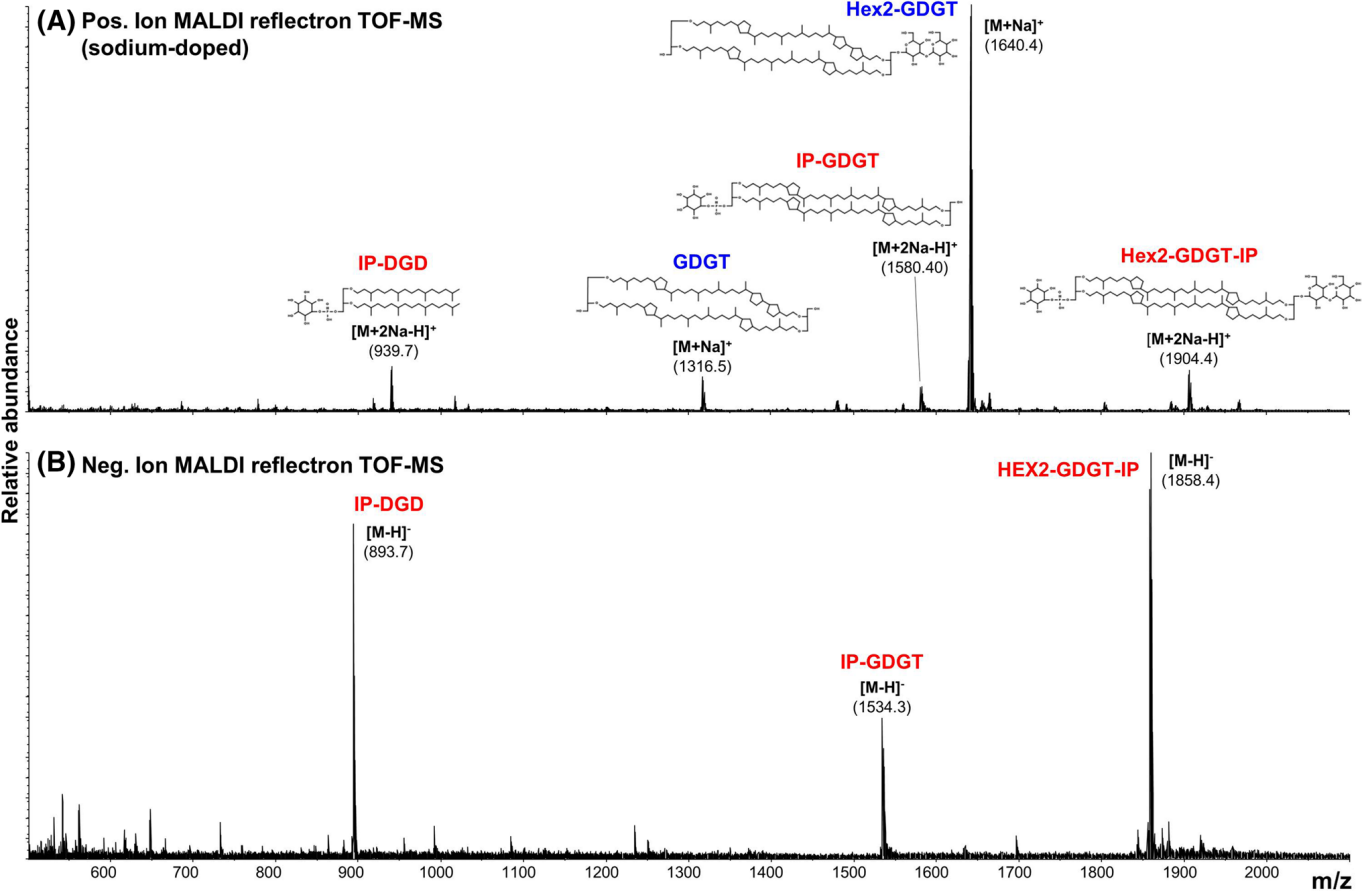
**a** Positive-ion MALDI reflectron TOF–MS spectrum of a *S. acidocaldarius* lipid extract spiked with NaCl. The spectrum shows the five most abundant lipid species in *S. acidocaldarius*. *m/z* values and structure of the dominant lipid species are given. **b** Negative-ion MALDI reflectron TOF–MS spectrum of the same sample. Noteworthy, in the negative-ion mode phosphate-free lipids (highlighted in blue in Fig. 2a) cannot be detected

Relative quantification of the lipid classes was performed based on peak height in the full scan mass spectra acquired in positive mode with NaCl spiking. Spiking with a surplus of NaCl guarantees uniform desorption/ionization of lipids and allows the detection of neutral lipids (Pittenauer and Allmaier 2009). For the comparison of the abundances we focused on the most abundant peaks in the lipid mass spectrum, exemplarily shown in Fig. 2. These were identified as the lipid classes Hex2–GDGT, IP–DGD, GDGT, IP–GDGT and Hex2–GDGT–IP.

**Fig. 3:**
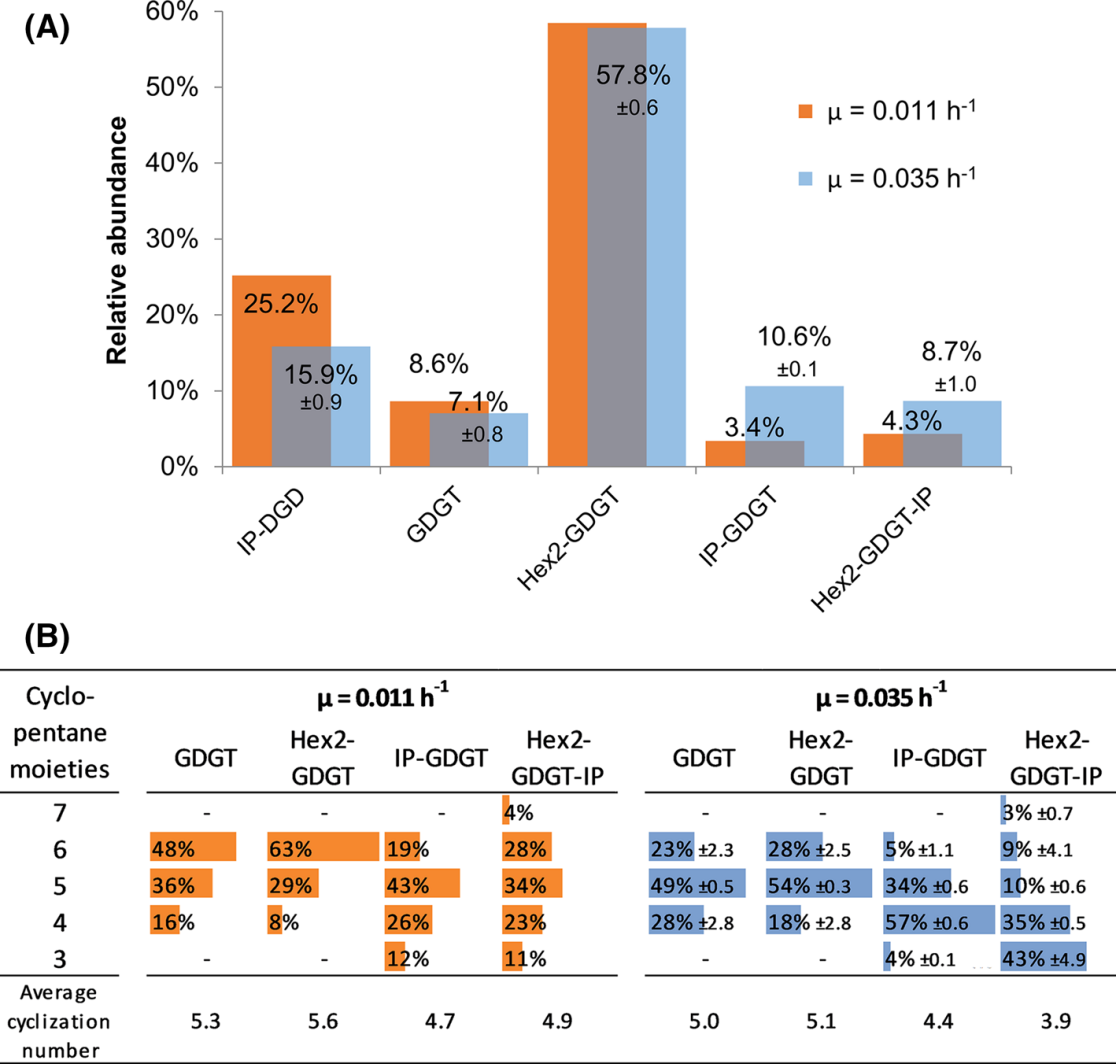
**a** Distribution of the major lipid classes in *S. acidocaldarius* in dependence of the growth rate (*µ*). Measurements were taken in positive MS mode with addition of NaCl. Values are given as % of the sum of major lipid classes. **b** Distribution of cyclopentane rings and average cyclization number in the investigated GDGT classes of *S. acidocaldarius* in dependence of *µ*. Errors indicated for the blue bars represent the divergence between the replicates of the two biological states (1 and 3) as shown in Fig. 1

The most abundant lipid class observed in *S. acidocaldarius* was Hex2–GDGT. Independent of the growth rate 58% of the total amount of lipids were Hex2–GDGT. As second most abundant class followed the diether IP–DGD with its share being highly dependent on the employed growth rate (25% at *µ* = 0.011 h^−1^ and 16% at *µ* = 0.035 h^−1^). IP–DGD was the only class of DELs that we found in significant amounts. GDGT, IP–GDGT and Hex2–GDGT–IP were present in amounts of 3–11% of total lipids. While the abundance of GDGT decreased from 9 to 7% when *µ* was increased, classes containing inositol phosphate were considerably more abundant at the higher *µ* (4% at *µ* = 0.011 h^−1^ and 9% at *µ* = 0.035 h^−1^). A summary of the results can be found in Fig. 3a.

The average cyclization number (De Rosa et al. 1980) was dependent on the employed *µ*. At the higher *µ* of 0.035 h^−1^ a minor fraction of the lipid species harbored six rings, while this share rose to approximately 36% when *µ* was decreased to 0.011 h^−1^. These distributions implicate average cyclization numbers of 5.1 at the lower *µ* and 4.6 at the higher *µ*. This inverse correlation between *µ* and average cyclization numbers was identified for all classes. Figure 3b gives an overview of the distribution of cyclopentane rings in the GDGT classes in dependence of *µ*.

In a recent investigation of the lipidomes of two *Sulfolobus* species, namely *S. islandicus* and *S. tokodaii*, Jensen et al. (2015b) used hybrid ion-trap orbitrap mass spectrometry in negative-ion mode to determine the lipid classes and lipid species in dependence of temperature (cultivation at optimal growth temperature and at ± 5 °C thereof) and growth phase (lag-, exponential- and stationary phase). These two species are by far the closest relatives to *S. acidocaldarius* that were investigated in terms of their membrane lipid composition. Based on 16S rDNA analysis *S. acidocaldarius* is phylogenetically more closely related to *S. tokodaii* (Quehenberger et al. 2017), while it is more similar to *S. islandicus* in regards to optimal growth temperature (75 °C for *S. acidocaldarius* and *S. islandicus* (Zhang et al. 2013) vs. 80 °C for *S. tokodaii* (Suzuki et al. 2002)). Table 2 shows the key indicators of the lipid composition which we will compare to the results of Jensen et al. (2015b) in the following paragraphs.

**Table 2:** Overview of key indicators of the lipid composition identified in this study

	*µ* = 0.011 h^−1^	*µ* = 0.035 h^−1^
Most abundant lipid classes	Hex2–GDGT–IP, Hex2–GDGT, IP–GDGT, GDGT and IP–DGD	
DEL:TEL ratio	1:3	1:5.5
Average cyclization number	5.1	4.6

### Abundance of different lipid classes

The results of the present study and the study by Jensen et al. are similar, with Hex2–GDGT–IP, IP–GDGT and IP–DGD being identified as major lipid classes in both studies. Additionally, we identified significant amounts of Hex2–GDGT or Hex–GDNT, two compounds that we were not able to distinguish with the used MS method. Jensen et al. also found significant amounts of sulfono-Hex3–GDGT–IP, while we detected this lipid class only in minor quantities in our samples. Possibly, this is a result of a sample preparation or measurement bias, causing an over- or underestimation of the compound. Also the fact that we investigated the lipid composition using positive-ion mode has an influence on the detected lipid pattern, since neutral lipids are easily overlooked when only negative-ion mode mass spectra are considered (see Fig. 2).

### Ratio of DEL:TEL among the different *Sulfolobus* species

Jensen et al. found DEL:TEL ratios in the exponential phase of 1:8 and 1:5 for *S. islandicus* (75 °C) and *S. tokodaii* (80 °C), respectively. For the lag phase ratios of 1:3 and 1:2 were calculated. It should be noted that the ratios calculated in the study of Jensen et al. are strongly fluctuating with culture conditions and oftentimes it is hard to find a clear trend or pattern in the DEL:TEL ratios. Based on our results, the DEL:TEL ratio of *S. acidocaldarius* at 75 °C is 1:3 for the lower *µ* of 0.011 h^−1^ and 1:5.5 for the higher *µ* of 0.035 h^−1^. These values lie within the ratios determined for the two related species.

### Number of cyclopentane moieties

According to the results of Jensen et al. when grown at the same temperature the lipids of *S. islandicus* have, on average, approximately one ring less compared to *S. tokodaii* (4 vs. 5 rings). A temperature shift of 5 °C results in a difference of ~ 0.5 rings for both species and since the optimal growth temperature of *S. islandicus* is 5 °C lower than for *S. tokodaii*, the two species actually differ by ~ 1.5 cyclopentane rings during the exponential phase at their respective optimal growth conditions. For *S. acidocaldarius* we found the average number of rings for the higher growth rate to be 4.6 at the optimal growth temperature of 75 °C.

### Effect of growth phase/growth rate

Jensen et al. showed for both organisms that the ring number dropped during the exponential phase and rose again in the stationary phase. They hypothesized that rather than the growth phase the specific growth rate might be the reason for the change in the lipid pattern. Indeed, while the correlation between average cyclization number and calculated growth rate was rather low for *S. islandicus* (0.43–0.15), in the case of *S. tokodaii* they observed a very high correlation (≥ −0.97). We found the same relationship for *S. acidocaldarius*. Jensen et al. “suspected the reason behind the observation in a lacking speed of lipid cyclization during biosynthesis at high growth rates”. Interestingly, in all three *Sulfolobus* species the number of rings decreases at the higher growth rate, while at the same time the share of TELs increases. A possible explanation for this behavior is that caused by the lack of nutrients the organism produces the chemically simpler archaeoles at the expense of the much more complex cyclopentane ring-bearing caldarchaeols (Jain et al. 2014; Villanueva et al. 2014). Subsequently, more cyclopentane rings are incorporated per remaining molecule of caldarchaeol.

## Conclusion

The lipid membrane of Archaea is a flexible and highly adaptive system. In this study we show that for the Crenarchaeum *S. acidocaldarius* this flexibility is not only a reaction of homeoviscous adaption to compensate for environmental factors, like shifts in pH or temperature, in order to maintain a constant state of membrane fluidity. Rather, *S. acidocaldarius* reacts with a change in the lipid composition to cope with nutrient limitation. The initial hypothesis maintains that *S. acidocaldarius* reacts to changes in *µ* with a change in its lipid pattern. More precisely, at a higher *µ* the share of IP–GDGT and Hex2–GDGT–IP increases at the expense of IP–DGD, while the average number of cyclopentane rings decreases. Since this study was performed in equilibrated cultivation conditions, these changes in the lipid pattern are truly consequences of a different growth rate and also happen in absence of the influence of different physiological states, as present in case of lag, exponential and stationary phase.

Summarizing, in this study we identified a novel factor (the controlled specific growth rate) for influencing the lipid composition of *S. acidocaldarius*. We were able to determine the effect of this factor on the lipid composition for the first time using MALDI-MS for the semi-quantification of an archaeal lipidome. A shift in the specific growth rate during a controlled continuous cultivation of *S. acidocaldarius* led to a change in the ratio of diether to tetraether lipids and in the average number of cyclopentane rings. Limitation of the growth rate led to an increase of the share of diethers, while the remaining tetraether lipids featured a higher average cyclization number.

## Electronic supplementary material

Below is the link to the electronic supplementary material.

Supplementary file1 (PDF 545 kb)

## References

[CR1] Belgacem O, Pittenauer E, Openshaw ME, Hart PJ, Bowdler A, Allmaier G (2016). Axial spatial distribution focusing: improving MALDI-TOF/RTOF mass spectrometric performance for high-energy collision-induced dissociation of biomolecules. Rapid Commun Mass Spectrom.

[CR2] Bischof LF, Haurat MF, Hoffmann L, Albersmeier A, Wolf J, Neu A (2019). Early response of *Sulfolobus acidocaldarius* to nutrient limitation. Front Microbiol.

[CR3] Chong PL-G (2010). Archaebacterial bipolar tetraether lipids: physico-chemical and membrane properties. Chem Phys Lipid.

[CR4] Damsté JSS, Schouten S, Hopmans EC, van Duin ACT, Geenevasen JAJ (2002). Crenarchaeol the characteristic core glycerol dibiphytanyl glycerol tetraether membrane lipid of cosmopolitan pelagic crenarchaeota. J Lipid Res.

[CR5] Damsté JSS, Rijpstra WIC, Hopmans EC, den Uijl MJ, Weijers JWH, Schouten S (2018). The enigmatic structure of the crenarchaeol isomer. Org Geochem.

[CR6] Dawson KS, Freeman KH, Macalady JL (2012). Molecular characterization of core lipids from halophilic archaea grown under different salinity conditions. Org Geochem.

[CR7] De Rosa M, Gambacorta A (1988). The lipids of archaebacteria. Prog Lipid Res.

[CR8] De Rosa M, Esposito E, Gambacorta A, Nicolaus B, BuLock JD (1980). Effects of temperature on ether lipid composition of *Caldariella acidophila*. Phytochemistry.

[CR9] Elling FJ, Könneke M, Lipp JS, Becker KW, Gagen EJ, Hinrichs K-U (2014). Effects of growth phase on the membrane lipid composition of the thaumarchaeon Nitrosopumilus maritimus and their implications for archaeal lipid distributions in the marine environment. Geochim Cosmochim Acta.

[CR10] Guan Z, Delago A, Nußbaum P, Meyer BH, Albers S-V, Eichler J (2017). Gene deletions leading to a reduction in the number of cyclopentane rings in *Sulfolobus acidocaldarius* tetraether lipids. FEMS Microbiol Lett.

[CR11] Jain S, Caforio A, Driessen AJM (2014). Biosynthesis of archaeal membrane ether lipids. Front Microbiol.

[CR12] Jensen SM, Brandl M, Treusch AH, Ejsing CS (2015). Structural characterization of ether lipids from the archaeon *Sulfolobus islandicus* by high-resolution shotgun lipidomics. J Mass Spectrom.

[CR13] Jensen SM, Neesgaard VL, Skjoldbjerg SLN, Brandl M, Ejsing CS, Treusch AH (2015). The effects of temperature and growth phase on the lipidomes of *Sulfolobus**islandicus* and *Sulfolobus**tokodaii*. Life.

[CR14] Kaneshiro SM, Clark DS (1995). Pressure effects on the composition and thermal behavior of lipids from the deep-sea thermophile *Methanococcus jannaschii*. J Bacteriol.

[CR15] Knappy CS, Chong JPJ, Keely BJ (2009). Rapid discrimination of archaeal tetraether lipid cores by liquid chromatography-tandem mass spectrometry. J Am Soc Mass Spectrom.

[CR16] Koga Y, Morii H (2007). Biosynthesis of ether-type polar lipids in archaea and evolutionary considerations. Microbiol Mol Biol Rev.

[CR17] Kramer JKG, Sauer FD (1991). Changes in the diether-to-tetraether-lipid ratio during cell growth in *Methanobacterium thermoautotrophicum*. FEMS Microbiol Lett.

[CR18] Lai D, Springstead JR, Monbouquette HG (2008). Effect of growth temperature on ether lipid biochemistry in *Archaeoglobus fulgidus*. Extremophiles.

[CR19] Matsuno Y, Sugai A, Higashibata H, Fukuda W, Ueda K, Uda I (2009). Effect of growth temperature and growth phase on the lipid composition of the archaeal membrane from *Thermococcus kodakaraensis*. Biosci Biotechnol Biochem.

[CR20] Meador TB, Gagen EJ, Loscar ME, Goldhammer T, Yoshinaga MY, Wendt J (2014). Thermococcus kodakarensis modulates its polar membrane lipids and elemental composition according to growth stage and phosphate availability. Front Microbiol.

[CR21] Morii H, Koga Y (1993). Tetraether type polar lipids increase after logarithmic growth phase of *Methanobacterium thermoautotrophicum* in compensation for the decrease of diether lipids. FEMS Microbiol Lett.

[CR22] Morii H, Eguchi T, Nishihara M, Kakinuma K, König H, Koga Y (1998). A novel ether core lipid with H-shaped C80-isoprenoid hydrocarbon chain from the hyperthermophilic methanogen *Methanothermus fervidus*. Biochim Biophys Acta-Lipids Lipid Metab.

[CR23] Oger PM, Cario A (2013). Adaptation of the membrane in Archaea. Biophys Chem.

[CR24] Pittenauer E, Allmaier G (2009). The renaissance of high-energy CID for structural elucidation of complex lipids: MALDI-TOF/RTOF-MS of alkali cationized triacylglycerols. J Am Soc Mass Spectrom.

[CR25] Quehenberger J, Shen L, Albers S-V, Siebers B, Spadiut O (2017). *Sulfolobus*—a potential key organism in future biotechnology. Front Microbiol.

[CR26] Quehenberger J, Albersmeier A, Glatzel H, Hackl M, Kalinowski J, Spadiut O (2019). A defined cultivation medium for *Sulfolobus acidocaldarius*. J Biotechnol.

[CR27] Shimada H, Nemoto N, Shida Y, Oshima T, Yamagishi A (2008). Effects of pH and temperature on the composition of polar lipids in *Thermoplasma acidophilum* HO-62. J Bacteriol.

[CR28] Suzuki T, Iwasaki T, Uzawa T, Hara K, Nemoto N, Kon T (2002). *Sulfolobus tokodaii* sp. nov. (f. *Sulfolobus* sp. strain 7), a new member of the genus *Sulfolobus* isolated from Beppu Hot Springs Japan. Extremophiles.

[CR29] van de Vossenberg JLCM, Driessen AJM, Konings WN (1998). The essence of being extremophilic: the role of the unique archaeal membrane lipids. Extremophiles.

[CR30] Villanueva L, Damsté JSS, Schouten S (2014). A re-evaluation of the archaeal membrane lipid biosynthetic pathway. Nat Rev Microbiol.

[CR31] Yoshinaga MY, Gagen EJ, Wörmer L, Broda NK, Meador TB, Wendt J (2015). *Methanothermobacter thermautotrophicus* modulates its membrane lipids in response to hydrogen and nutrient availability. Front Microbiol.

[CR32] Zhang C, Cooper TE, Krause DJ, Whitaker RJ (2013). Augmenting the genetic toolbox for *Sulfolobus islandicus* with a stringent positive selectable marker for agmatine prototrophy. Appl Environ Microbiol.

