# Supplementary material for: The influence of the specific growth rate on the lipid composition of Sulfolobus acidocaldarius
Source: Extremophiles. 2020 Mar 21;24(3):413–20. doi: 10.1007/s00792-020-01165-1 (PMC7174258; doi:10.1007/s00792-020-01165-1)
Supplement: Supplementary file 1 — Supplementary file1 (PDF 545 kb) [file 792_2020_1165_MOESM1_ESM.pdf]

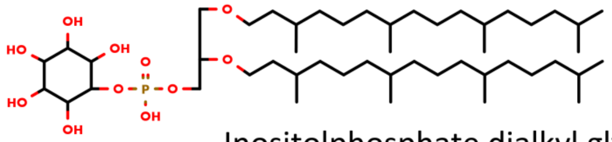

Inositolphosphate dialkyl glycerol diether (**IP-DGD**)

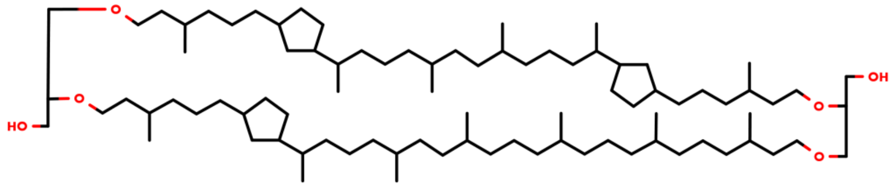

Glycerol dialkyl glycerol tetraether (**GDGT**)

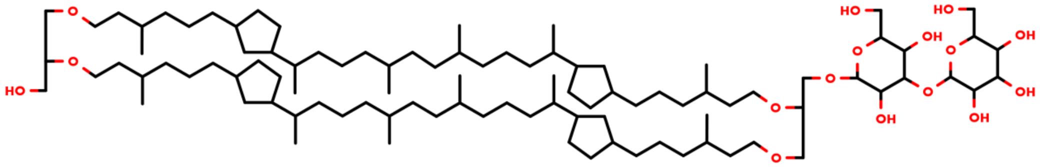

Dihexose glycerol dialkyl glycerol tetraether **(Hex2-GDGT**)

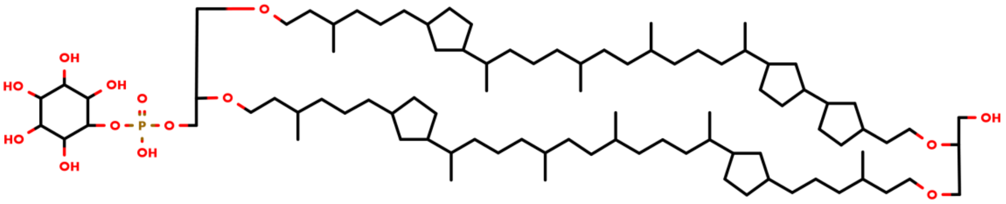

Inositolphosphate glycerol dialkyl glycerol tetraether (**IP-GDGT**)

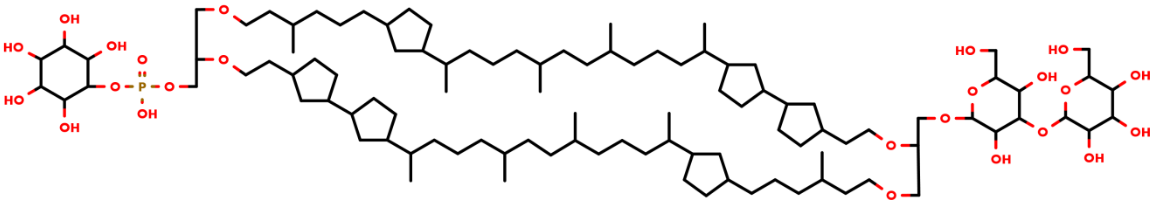

Dihexose glycerol dialkyl glycerol tetraether inositolphosphate **(Hex2-GDGT-IP**)

Supplementary Fig. 1

Structures of the most abundant lipid classes in *Sulfolobus acidocaldarius*. The number of cyclopentane rings depicted is exemplarily and generally vary for all glycerol dialkyl glycerol tetraethers (GDGTs) from 3 to 6 per lipid. The position of cyclopentane rings was not determined in this study. Also note that with the used MS method we were not able to distinguish between GDGT and GDNT. Structures were drawn with MarvinSketch 19.3.0, 2019, ChemAxon
